# Primary breast tumor induced extracellular matrix remodeling in premetastatic lungs

**DOI:** 10.1038/s41598-023-45832-7

**Published:** 2023-10-30

**Authors:** Ruoqing Cai, Caitlin M. Tressler, Menglin Cheng, Kanchan Sonkar, Zheqiong Tan, Santosh Kumar Paidi, Vinay Ayyappan, Ishan Barman, Kristine Glunde

**Affiliations:** 1 The Russell H. Morgan Department of Radiology and Radiological Science, Division of Cancer Imaging Research, The Johns Hopkins University School of Medicine, 720 Rutland Avenue, Traylor Building, Room 203, Baltimore, MD 21205 USA; 2 The Sidney Kimmel Comprehensive Cancer Center, The Johns Hopkins University School of Medicine, Baltimore, MD USA; 3 Department of Mechanical Engineering, Johns Hopkins University, Baltimore, MD USA; 4 Department of Biological Chemistry, Johns Hopkins University School of Medicine, Baltimore, MD USA; 5 Department of Medical Laboratory, The Central Hospital of Wuhan, Tongji Medical College, Huazhong University of Science and Technology, Wuhan, Hubei China

**Keywords:** Cancer, Breast cancer, Cancer imaging, Cancer microenvironment, Metastasis

## Abstract

The premetastatic niche hypothesis proposes an active priming of the metastatic site by factors secreted from the primary tumor prior to the arrival of the first cancer cells. We investigated several extracellular matrix (ECM) structural proteins, ECM degrading enzymes, and ECM processing proteins involved in the ECM remodeling of the premetastatic niche. Our in vitro model consisted of lung fibroblasts, which were exposed to factors secreted by nonmalignant breast epithelial cells, nonmetastatic breast cancer cells, or metastatic breast cancer cells. We assessed ECM remodeling in vivo in premetastatic lungs of female mice growing orthotopic primary breast tumor xenografts, as compared to lungs of control mice without tumors. Premetastatic lungs contained significantly upregulated Collagen (Col) Col4A5, matrix metalloproteinases (MMPs) MMP9 and MMP14, and decreased levels of MMP13 and lysyl oxidase (LOX) as compared to control lungs. These in vivo findings were consistent with several of our in vitro cell culture findings, which showed elevated Col14A1, Col4A5, glypican-1 (GPC1) and decreased Col5A1 and Col15A1 for ECM structural proteins, increased MMP2, MMP3, and MMP14 for ECM degrading enzymes, and decreased LOX, LOXL2, and prolyl 4-hydroxylase alpha-1 (P4HA1) for ECM processing proteins in lung fibroblasts conditioned with metastatic breast cancer cell media as compared to control. Taken together, our data show that premetastatic priming of lungs by primary breast tumors resulted in significant ECM remodeling which could facilitate metastasis by increasing interstitial fibrillar collagens and ECM stiffness (Col14A1), disruptions of basement membranes (Col4A5), and formation of leaky blood vessels (MMP2, MMP3, MMP9, and MMP14) to promote metastasis.

## Introduction

Breast cancer is the most common form of cancer in women. The survival rate drops drastically in Stage IV breast cancer patients who have metastatic nodules in the lungs, which is one of the most common sites of systemic metastases in late-stage solid cancers^1,2^. The molecular mechanisms of metastasis which modulate the microenvironment at distant organ sites and thereby enable the engraftment of circulating tumor cells, remain elusive. The idea of processes that prime the metastatic site prior to the arrival of the first cancer cells, was first described by Stephen Paget in 1889 in his “seed and soil” hypothesis^3^. Later studies revealed that factors which are secreted by the primary tumor, indeed determine the organ site of metastasis and lead to molecular changes which support the survival and growth of the arriving metastatic cancer cells^4–6^. Such molecularly altered microenvironments in future metastatic organ sites, which can be observed prior to the arrival of the first cancer cells, have been termed premetastatic niches^4–6^. Increased levels of inflammatory chemokines, matrix metalloproteinases (MMPs), and adhesion molecules have been observed in premetastatic niches^7–9^. The recruitment of pro-tumor immune cells, such as neutrophils, monocytes, and macrophages, as well as active, continuous remodeling of the extracellular matrix (ECM) has been detected in premetastatic niches^7–9^. Particularly, specific ECM changes in the premetastatic niche play a significant role in creating a supportive environment for cancer cell growth in the metastatic site^7–9^. Moreover, during metastatic colonization, cancer cells can induce an inflammatory phenotype in lung fibroblasts which increases the growth of lung metastases and may participate in the remodeling of the ECM in the premetastatic niche^10^.

In breast cancer, increased expression levels of fibrillar collagens-1 and -3 were shown to be linked to tumor invasion and aggressiveness^11,12^. In our previous studies, collagen-1 fiber densities increased in both metastatic lymph nodes and premetastatic lungs in an orthotopic mouse xenograft model of highly metastatic breast cancer, which was compared to an orthotopic mouse xenograft model of nonmetastatic breast cancer^13,14^. The degradation of collagen-4 was also observed in the premetastatic niche, which promoted the recruitment of bone marrow-derived cells (BMDCs), which in turn created an environment permissive for the subsequent invasion and growth of tumor cells^15,16^. Considering the vital role of collagens in the formation of the lung premetastatic niche, several enzymes that participate in collagen synthesis were also shown to be critically involved in the formation of the premetastatic niche. Lysyl oxidase (LOX), which crosslinks collagen-1, was demonstrated to be secreted by primary breast cancer cells and accumulated in the premetastatic niche, especially under hypoxic conditions, as LOX is induced by hypoxia-inducible factor 1^16^. Prolyl-4-hydroxylase (P4HA) which catalyzes proline hydroxylation of procollagen-1 to allow for proper protein folding into stable triple helixes prior to collagen-1 secretion, was shown to be critically involved in premetastatic collagen-1 deposition, and P4HA inhibition decreased metastasis^17^. MMPs which degrade and remodel the extracellular matrix, have been demonstrated to play an important role in cancer invasion, angiogenesis, and overall cancer progression^15^. MMPs were also shown to participate in the formation of the premetastatic niche, as MMP2 degrades collagen-4^15^. Moreover, MMP3 and MMP10 expression levels have been associated with increased vascular permeability in the lungs, which in turn allowed for the extravasation of cancer cells to form lung metastases^18^.

In this study, we have investigated the expression profiles of proteins which could be involved in the ECM remodeling of premetastatic lungs. To specifically probe ECM-related premetastatic lung changes at the molecular level, we designed a cell culture model of human lung fibroblasts which were conditioned by secreted factors of differentially metastatic human breast cancer cells. This cell culture modeling approach, in conjunction with a mouse model of premetastatic niche formation in the lung^14^, was utilized to characterize consistent changes in ECM structural proteins, ECM degradome, and ECM processing proteins. Our studies revealed that premetastatic lung priming by primary breast tumors significantly remodeled the lung ECM through the modulation of ECM structural proteins, upregulation of ECM degrading enzymes, and downregulation of ECM processing proteins, which supported the premetastatic niche hypothesis.

## Results

### Breast cancer cell-induced ECM-related changes in molecular profiles of lung fibroblasts

The premetastatic niche hypothesis suggests that cancer cells in primary tumors secrete factors that travel through the blood stream and reach secondary organs in which they induce ECM changes at future metastatic sites. We tested if this hypothesis holds true in terms of primary tumor cells modifying fibroblast expression profiles in premetastatic lungs. To this end, we first compared the effects of conditioned media from breast epithelial cell lines of varying metastatic potential on lung fibroblasts in culture. The panel of human cell lines we used consisted of immortalized nonmalignant MCF12A breast epithelial cells, nonmetastatic MCF7 breast cancer cells and metastatic MDA-MB-231 breast cancer cells for generating conditioned media, in which normal human MRC5 lung fibroblast cells were incubated. We incubated MRC5 fibroblasts with fresh unconditioned media as control. This approach simulated the long-term exposure of lung fibroblasts to circulating factors that are secreted and released by primary breast tumors of varying metastatic potential. To investigate the expression profile changes of these four experimental groups of lung fibroblasts post-incubation, we performed qRT-PCR analyses of several mRNA expression levels from these fibroblasts as described in the following. Building on our previous data^13,14,19–22^, we focused this study on ECM related genes, and observed significant changes in several ECM structural proteins, MMPs, and ECM processing proteins.

**Figure 1:**
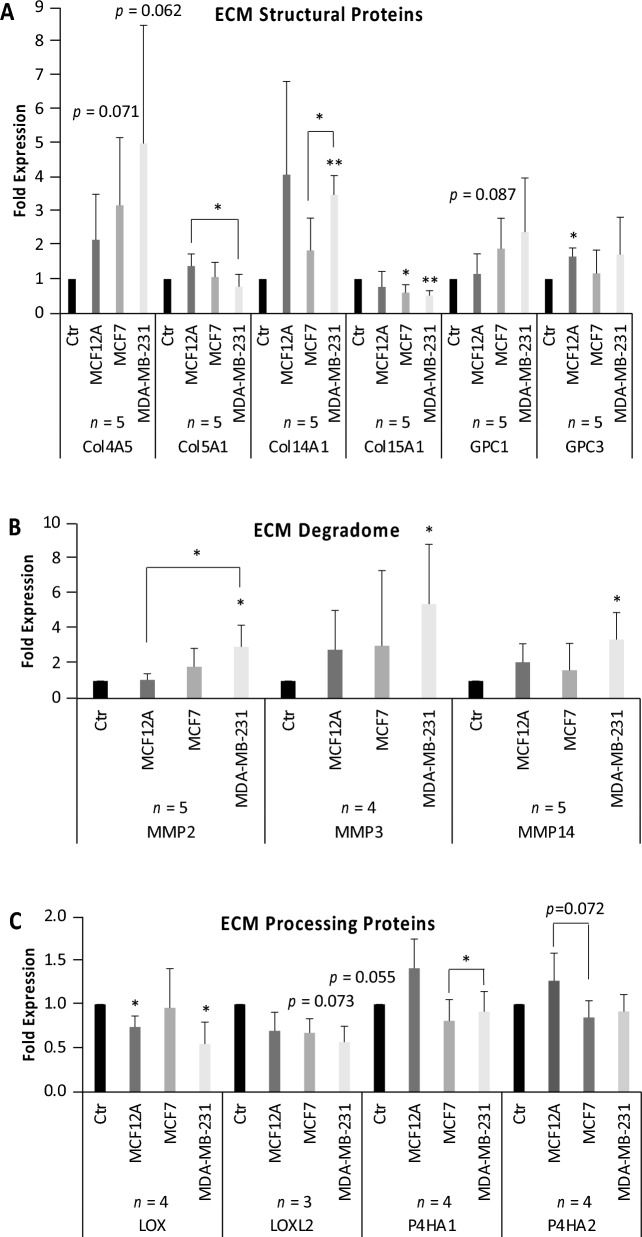
**Cell culture study.** Lung fibroblasts exposed to conditioned media from nonmalignant MCF12A breast epithelial cells, nonmetastatic MCF7 and metastatic MDA-MB-231 breast cancer cells for 24 h. (**A**) mRNA expression levels of ECM structural proteins in lung fibroblasts. Other mRNA levels tested include Col1A1, Col1A2, Col4A2, Col6A1 and GPC4, which did not show any significant changes. Col6A2, Col8A1, Col11A2, fibronectin (FN), version (VCAN) and hyaluronan synthase 2 (HAS2) were tested and showed a trend of increased expression in MDA-MB-231-conditioned fibroblasts. (**B**) mRNA expression levels of ECM degrading enzymes in lung fibroblasts. Other mRNA levels tested include MMP1, MMP10 and MMP25, which showed a trend of increased expression in MDA-MB-231-conditioned fibroblasts. Some mRNA levels, including MMP7, MMP8, MMP9 and MMP13, were too low for detection or accurate quantification. (**C**) mRNA levels of ECM processing proteins in lung fibroblasts. Other mRNA levels tested include hypoxia-inducible factor 1A (HIF1A) and lysyl oxidase like 4 (LOXL4), which did not show any significant changes.

Expression levels of the ECM structural proteins Col5A1 and Col15A1 significantly decreased while Col14A1 significantly increased in lung fibroblasts exposed to factors from metastatic breast cancer cells as compared to controls (Fig. 1A). Media conditioning with the nonmalignant cell line MCF12A had no significant effect on the lung fibroblast expression level of Col14A1, as the error bars were large due to large variability of the data. We also observed increased levels of Col4A5 and glypican (GPC) GPC1 in lung fibroblasts exposed to conditioned media from both breast cancer cells, which approached statistical significance. We detected a significant GPC3 increase in the lung fibroblasts conditioned with nonmalignant MCF12A media, but the increases for cancer cell conditioning did not reach significance.

Expression levels of ECM degrading proteases MMP2, MMP3, and MMP14 significantly increased in lung fibroblasts exposed to media conditioned by metastatic breast cancer cells as compared to controls (Fig. 1B, Supplementary Figure 1). MMP1 and MMP10 expression levels did not change significantly (Supplementary Figure 1).

We further tested several expression levels of key genes involved in the production of mature collagen fibers and observed significant decreases in LOX expression levels in lung fibroblasts exposed to media conditioned by MCF12A and MDA-MB-231 cells. For lysyl oxidase like (LOXL) LOXL2, none of the measured decreases for any of the three breast cell lines tested resulted in significant changes, but rather in trends toward significance for conditioning by MCF7 (p = 0.073) and MDA-MB-231 (p = 0.055) cells, but not by MCF12A cells. For P4HA1, no significant changes were observed for comparisons to control or MCF12A conditioned lung fibroblasts, just trends towards downregulation, but we detected a significant increase in P4HA1 in lung fibroblasts conditioned by metastatic MDA-MB-231 cells as compared to nonmetastatic MCF7 cells (Fig. 1C). For P4HA2, changes in expression levels did not reach significance, but MCF7 conditioned lung fibroblasts were trending towards significance for downregulation in P4HA2 as compared to MCF12A conditioned lung fibroblasts (Fig. 1C). Hyaluronan synthase 2 (HAS2) expression levels did not change significantly (Supplementary Figure 1).

Taken together, our cell culture studies show that breast cancer cells, and in particular metastatic breast cancer cells, without physical contact and solely through secreted factors, were able to affect gene expression levels in lung fibroblasts that would alter the deposition, degradation, and crosslinking of the ECM.

### Mouse studies of primary breast tumor-induced premetastatic changes in the lung ECM

**Figure 2:**
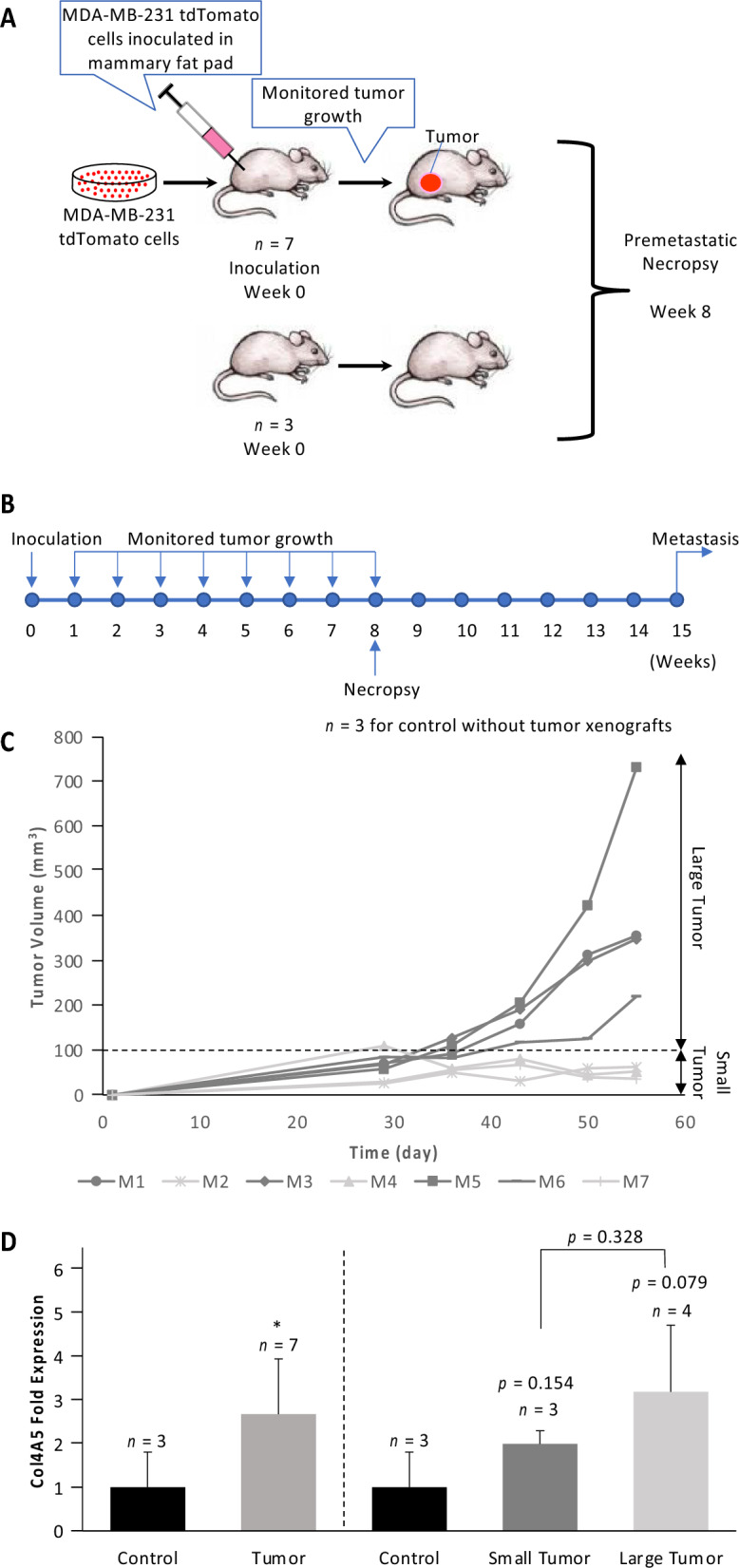
**Mouse study.** (**A**) Schematic overview of experimental design of premetastatic niche mouse study. Seven mice were orthotopically inoculated with MDA-MB-231-tdTomato cells. Three mice remained un-inoculated, serving as control mice without tumors. All mice are sacrificed after eight weeks. (**B**) Timeline of mouse study. Cells were inoculated at week 0, tumor growth was monitored weekly between week 0 and week 8, and premetastatic necropsy was performed at week 8, well prior to first occurrence of metastasis at week 15 (as per^14^). (**C**) Tumor growth curves of the seven inoculated mice. For some analyses, mice were grouped in two groups of large tumor > 100 mm^3^ and small tumor < 100 mm^3^, using 100 mm^3^ as tumor size cutoff. (**D**) mRNA levels of Col4A5 in homogenized lungs of both normal control mice and MDA-MB-231-tdTomato tumor-bearing mice. Other mRNA levels tested include Col1A2, Col4A3, Col5A1, Col15A1, FN1 and GPC4, which did not show any significant changes. We also tested Col14A1, GPC1, GPC3, HAS2, MMP1A, MMP1B, MMP2, MMP7, MMP8, MMP9, MMP10, MMP13 and MMP14 mRNA levels, which all showed a trend of progressively increasing expression in lungs of small and large tumor bearing mice as compared to normal control mice. We further tested LOX which showed a trend of decreased expression in the lungs of tumor-bearing mice compared to control mice.

To test if similar trends would hold true in more complex biological models, we orthotopically inoculated MDA-MB-231-tdTomato breast cancer cells into the mammary fat pads of seven athymic female nude mice (Fig. 2A). Three uninoculated mice served as controls (Fig. 2A). After 56 days (8 weeks) of tumor growth, mice were sacrificed prior to metastasis. This timeline was previously established in our lab and was repeatedly confirmed to not result in any metastatic nodules in the lungs^14,22^. The premetastatic status of all lungs was confirmed by in vivo fluorescence imaging, which showed that there was no tdTomato fluorescence detected from potential MDA-MB-231-tdTomato cells in any of the mouse lungs (Supplementary Figure 2). Owing to the variability in tumor sizes, we pursued a twofold data analysis approach, analyzing data pooled from all seven tumor-bearing mice, as well as divided into two groups of small and large tumor sizes, using 100 mm^3^ as size cutoff (Fig. 2C). We performed qRT-PCR analysis from homogenized lung tissues for the same mRNA expression levels of genes as in our cell culture studies shown in Fig. 1. Consistent with our cell culture data, Col4A5 was significantly upregulated in the lungs of tumor-bearing mice, especially in the lungs of mice with large tumor sizes (Fig. 2D). We also observed similar trends of mRNA expression levels in lungs of tumor-bearing mice (Supplementary Figures 3-4) in the same genes that exhibited significant, or close to significant, upregulation in MDA-MB-231 conditioned fibroblasts in our cell culture studies, namely Col14A1, GPC1, GPC3, HAS2, MMP1, MMP2, MMP10 and MMP14.

### Differential MMP protein expression in premetastatic versus normal lungs

**Figure 3:**
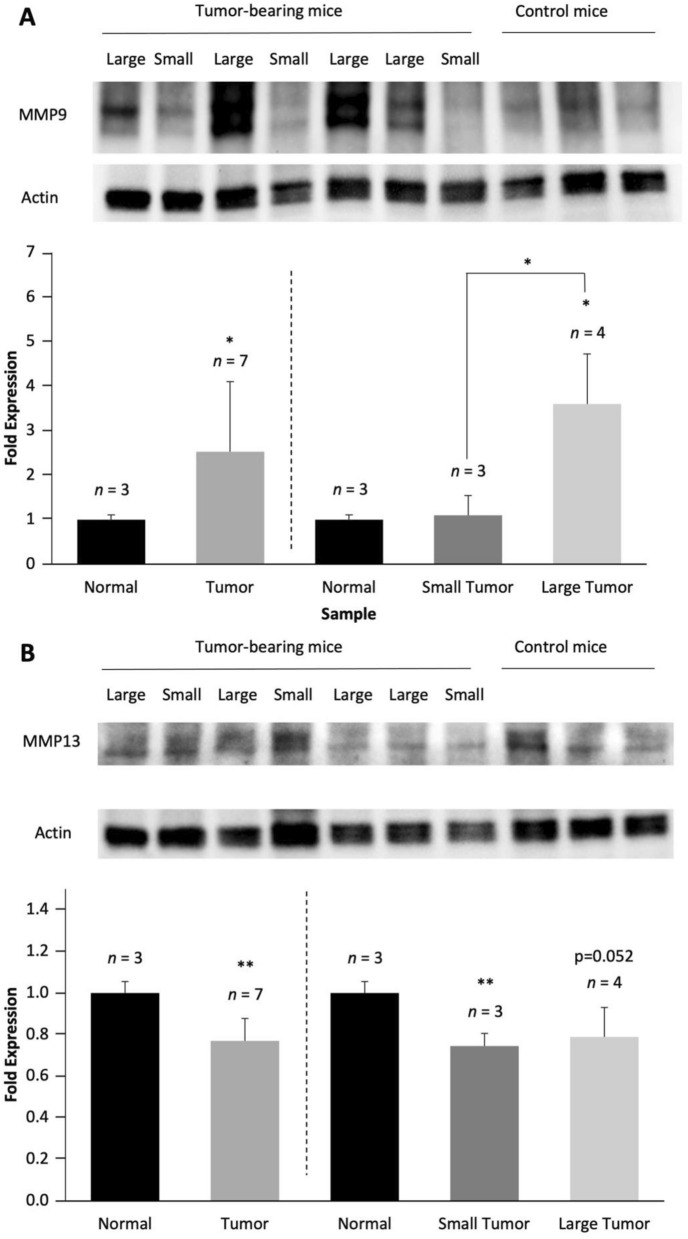
**Mouse study.** Lung tissues from all mice were homogenized individually and whole protein was isolated from each lung. Owing to the variability in tumor sizes, we pursued a twofold data analysis approach, analyzing data pooled from all seven tumor-bearing mice, as well as divided into two groups of small and large tumor sizes, using 100 mm^3^ as size cutoff (see Fig. 2C). (**A**) MMP9 protein expression levels were significantly increased in lungs of metastatic tumor-bearing mice *versus* control lungs. Western blots of MMP2 showed a similar trend (Supplementary Figure 5). (**B**) MMP13 protein expression levels were significantly decreased in lungs of metastatic tumor-bearing mice as compared to controls. Western blots of Col1 showed a similar trend (Supplementary Figure 6). Western blots of MMP7 and MMP14 showed no significant changes between normal control lungs and lungs from tumor-bearing mice.

To further evaluate ECM-related changes at the protein level, we performed Western blot analyses of collagen-1 and several key MMPs which exhibited trends of mRNA upregulation in premetastatic lungs of metastatic breast tumor bearing mice *versus* control mice. Consistent with the corresponding mRNA expression results, collagen-1 showed a trend of decreased expression in the lungs of metastatic breast tumor-bearing mice, especially in large tumors (Supplementary Figure 5). In terms of the ECM degradome, corresponding to their trend of upregulation at the mRNA level, some MMPs exhibited consistent trends of protein upregulation in premetastatic lungs of metastatic breast tumor-bearing mice compared to control lungs. Specifically, our protein expression data showed both a trend of increased MMP2 levels (Supplementary Figure 5) and a significant increase in MMP9 levels in lungs from mice growing orthotopic MDA-MB-231-tdTomato tumor xenografts compared to lungs from normal mice as controls (Fig. 3A). MMP9 upregulation was more pronounced in large tumors as compared to small tumors, suggesting a positive correlation between lung MMP9 protein expression level and tumor size (Fig. 3A). Despite a trend of upregulation in mRNA level, MMP13 exhibited a significant decrease in the premetastatic lungs as compared to control lungs, particularly in small tumors (Fig. 3B). MMP7 and MMP14 did not show any significant changes at the protein level even though their mRNA levels showed trends of progressive increases in lungs of tumor-bearing mice with increasing tumor sizes (Supplementary Figure 4 and 5).

### ECM structural proteins, ECM degradome and ECM processing proteins in premetastatic versus normal lungs

We observed premetastatic changes in three classes of ECM-related proteins, i.e., ECM structural proteins, ECM degradome, and ECM processing proteins, which were consistent in cell culture studies of lung fibroblasts conditioned by factors secreted by breast cancer cells as well as in premetastatic lungs of breast tumor bearing mice. For ECM structural proteins, we found consistent trends of moderate upregulation of Col4A5, Col14A1, GPC1, GPC3 and HAS2 (Figs. 1, 2, Supplementary Figure 1, 3). Among these proteins, expression levels of Col4A5, GPC1 and HAS2 increased progressively with metastatic potential or tumor size in cell and animal experiments, respectively (Figs. 1, 2, Supplementary Figure 1, 3). Some ECM structural proteins, including Col5A1 and Col15A1, were downregulated in the cancer-conditioned fibroblasts in our cell culture experiments (Fig. 1). Yet, these changes were not detected in premetastatic lungs in animal studies, especially for larger tumors (Supplementary Figure 3). For the matrix degradome, MMP1, MMP2, MMP10 and MMP14 showed trends of progressive upregulation with increasing metastatic potential in lung fibroblasts exposed to secreted factors from breast cancer cells (Fig. 1, Supplementary Figure 1). Increases in MMP1, MMP7 and MMP13 expression levels in premetastatic lungs as compared to normal lungs (Fig. 3, Supplementary Figure 4, 5) were more extensive than those of ECM structural proteins. For ECM processing proteins, LOX was consistently downregulated across all experiments in premetastatic, cancer-conditioned groups versus controls (Fig. 1, Supplementary Figure 3).

## Discussion

**Figure 4:**
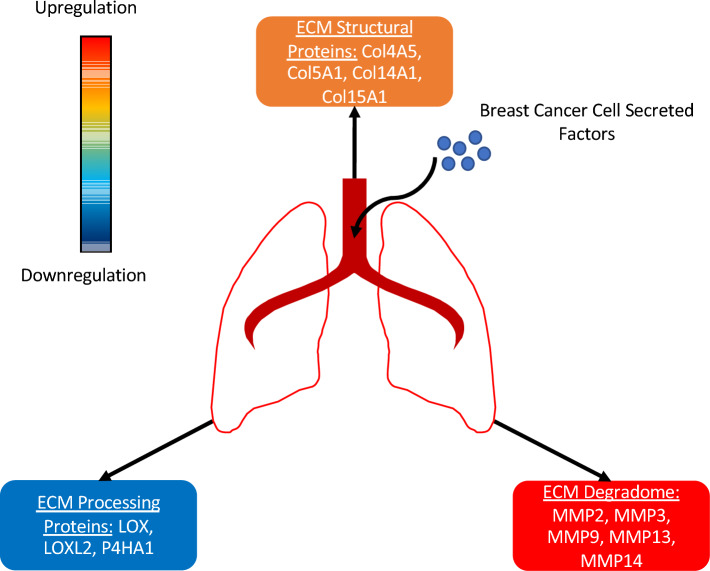
**Summary of findings.** Premetastatic pulmonary changes were observed in three classes of ECM-related proteins: moderate upregulation of ECM structural proteins, significant upregulation of ECM degradome (ECM degrading enzymes), and moderate downregulation of ECM processing protein.

In this study, we have clearly shown that primary metastatic breast tumors were able to modulate the expression levels of ECM structural proteins (Col14A1, Col4A5, Col5A1, Col15A1), ECM degrading enzymes (MMP2, MMP3, MMP9, MMP13, and MMP14), and ECM processing proteins (LOX, LOXL2, P4HA1) in premetastatic lungs prior to the arrival of the first cancer cells. In summary, we observed upregulation in ECM-degrading enzymes and downregulation of ECM processing proteins, which resulted in a moderate overall increase in ECM structural proteins in both cell and animal experiments (Fig. 4). Our data suggest that this was, at least partially, mediated by lung fibroblasts which were stimulated by factors secreted from breast cancer cells in the primary tumor. Our findings support the premetastatic niche hypothesis^1,2,4–6^, as well as previous findings that the ECM is significantly altered in metastatic target organs after the arrival of cancer cells^13,14,20^.

We observed premetastatic lung increases in Col14A1, which encodes a subunit of collagen-14^23^, and which was consistent in premetastatic lungs of mice growing primary breast tumors, as well as lung fibroblasts exposed to metastatic cancer-conditioned media. This finding is in good agreement with a previous study showing elevated collagen-14 levels in lymph node metastases compared to non-metastatic tissues, suggesting its involvement in metastasis^23^. Our work shows for the first time that collagen-14 remodeling likely occurs prior to the arrival of metastatic cancer cells in the lungs. The elevated Col14A1 in premetastatic lungs which was observed in our study, may aid metastasis by stimulating fibrillogenesis, which is a main function of collagen-14, thereby increasing interstitial fibrillar collagens and ECM stiffness to promote metastasis. Our study also revealed that primary breast tumors stimulated increases in Col4A5 in premetastatic lungs and breast cancer-conditioned lung fibroblasts, which was consistent in our cell culture and animal studies. Col4A5 encodes a subunit of collagen-4^24^. Collagen-4 increase and remodeling in premetastatic lungs, which was observed in our study, could play an important role in disruptions of basement membranes to facilitate metastasis formation in the lungs, as collagen-4 is an integral part of basement membranes^11^.

In addition, several MMPs that contribute to the proteolysis of collagen-4^25,26^, including MMP2, MMP3, MMP9, and MMP14, were upregulated in either lung fibroblasts exposed to breast cancer cell-conditioned media, or premetastatic lungs in metastatic primary tumor-bearing mice. MMP13 was significantly downregulated in premetastatic lungs. Our results suggest that cancer cells in primary breast tumors release factors that stimulate premetastatic lung fibroblasts to remodel the collagen-4 matrix in lungs by means of laying down more collagen-4 and degrading pre-existing collagen. This finding is consistent with previous findings of elevated MMP9 levels in the premetastatic niche along with collagen-4 degradation mediated by MMP2 secreted from BMDCs^15^. MMP changes in the premetastatic niche were previously shown to have several functions. In agreement with our data, MMP9 was reported to be secreted by lung-residing monocytic myeloid-derived suppressor cells (M-MDSC) prior to metastasis to enhance the lung’s ability of tumor cell entrapment^27^. MMP9 expression levels correlated with immune cell recruitment in the lungs prior to metastasis^28^, as well as with the formation of leaky vasculature in premetastatic lungs in breast tumor-bearing mice^29^. Also in agreement with our study, MMP3 was reported to be involved in premetastatic remodeling of lung blood vessels and escape of circulating tumor cells in a murine melanoma tumor model^18^. Taken together, the MMP2, MMP3, MMP9, and MMP14 upregulation observed in premetastatic lungs in our study may enable metastasis by immune cell recruitment and remodeling of the lung vasculature to form leaky blood vessels which facilitate metastasis.

Our study revealed consistent downregulation of the matrix processing protein LOX in premetastatic lungs as well as in breast cancer-conditioned lung fibroblasts. Previous studies have shown that LOX increased in premetastatic sites in the lungs through increased expression and secretion from breast cancer cells in the primary tumor, which was induced by hypoxia-inducible factor 1 alpha (HIF1-$\alpha$) under hypoxic conditions^16^. Elevated expression of LOX was also observed in premetastatic lungs of mice growing metastatic primary SUM159 tumors compared with lungs of mice growing nonmetastatic MCF7 tumors^30^. These two previous studies and our data used different tumor models and vastly different tumor sizes, i.e., average size of 260 mm^3^ in our study in MDA-MB-231-tdTomato tumors and 1500 mm^3^ in SUM159 tumors in^30^. This could explain the reduction in LOX levels in the premetastatic lungs in our study, rather than LOX increase as reported in^16,30^, as hypoxic regions in large primary tumors were discussed as a major cause of premetastatic lung LOX increase and our smaller primary tumors may have a relative lack of LOX expression^16^. In addition, collagen-4 is a substrate for LOX which promotes the recruitment of BMDCs to the premetastatic niche^15^. Our data are also in agreement with a recent in vitro study which showed that tumor cell-conditioned media drive collagen remodeling through fibroblast and pericyte activation^31^.

Lastly, we would like to mention some of the limitations of our study, which can have a potential impact on its outcomes. These limitations include the limited number of cell lines studied (three cell lines), the limited number of animals per group (seven premetastatic animals, three control animals), and the variability in primary tumor sizes (partially mitigated by separately analyzing large versus small tumor sizes). Our findings that nonmalignant MCF12A cells in cell culture were able to induce a significant increase in GPC3 and a significant decrease in LOX in lung fibroblasts may be explained by the limited number of breast cell lines used and was also not consistent with our in vivo findings in our premetastatic lung niche animal model.

In summary, our study revealed that primary metastatic breast tumors release factors that alter the expression levels of ECM structural proteins, ECM degrading enzymes, and ECM processing proteins in premetastatic lungs, which was mediated, at least in part, by lung fibroblasts, supporting the premetastatic niche hypothesis^1,2,4–6^, and ECM alterations in metastatic sites^13,14,20^. These findings confirm the involvement of lung fibroblasts in the establishment of the premetastatic niche and their role in modulating the ECM to provide a fertile microenvironment for cancer cell engraftment. The involvement of fibroblasts in the progression of metastasis may be evolving dynamically over time and may grow more pronounced from premetastatic, to micro-metastatic, to macro-metastatic stages^10^.

## Methods

### Cell culture

We used human immortalized normal MCF12A breast epithelial cells, human nonmetastatic MCF7 breast cancer cells, and human metastatic MDA-MB-231 breast cancer cells, all of which were purchased from the American Type Culture Collection (ATCC, Manassas, VA). All breast cell lines were cultured under sterile conditions without antibiotics as previously described until they reached 80% confluence^19^. Normal human MRC-5 lung fibroblasts were purchased from ATCC and cultured in the same media as MCF7 cells, which was Minimum Essential Media (MEM, ThermoFisher Scientific, Waltham, MA) with 10% fetal bovine serum (FBS). All cell lines were free of mycoplasma and authenticated by short tandem repeat (STR) profiling. All cell lines were grown and incubated in a humidified atmosphere at 5% CO_2_. When breast cell lines reached 80% confluence, media was changed to MEM with 10% FBS, which was collected after 24 h of incubation. These conditioned media contained all secreted factors that the respective breast cells had released into the media over 24 h. Lung fibroblasts at 40% confluence were then incubated with these conditioned media for 72 h. Control fibroblasts were incubated with normal media without prior conditioning by any cells. For cell harvesting, all cells were rinsed twice with phosphate-buffered saline (PBS, Corning, Manassas, VA), suspended using buffer as detailed below, and scraped off with a cell scraper.

### Animal experiments

The Johns Hopkins Medical Institutions are fully accredited by the American Association for the Accreditation of Laboratory Animal care (AAALAC). The conduct and reporting of the described experiments adhere to the principles enumerated in the “A Guide to the Care & Use of Laboratory Animals” prepared by the Committee on Care & Use of Laboratory Animals of the Institute of Laboratory Animal Resources (ILAR), National Research Council (NRC) (International Standard Book, Number 0–309-05,377–3, 1996). All procedures were also performed in accordance with the Health Resource Extension Act of 1985, Public Law 99–158 (1985). The Principles for Laboratory Animal Research outlined by the American Physiological Society were also followed. All animal experiments were approved by the Institutional Animal Care and Use Committee (IACUC) of the Johns Hopkins University School of Medicine. The reporting in the manuscript follows the recommendations in the ARRIVE guidelines. Ten adult female athymic nude nu/nu mice (Taconic Biosciences, Rensselaer, NY) were orthotopically inoculated into the 4th mammary fat pad with two million MDA-MB-231-tdTomato cells, which constitutively express the tdTomato fluorescent protein to allow for monitoring of lung metastasis in vivo^14^. Tumor sizes were measured weekly with calipers starting at four weeks post-inoculation when tumors were palpable. The absence of metastasis was confirmed by the absence of tdTomato-fluorescing breast cancer cells in all mouse lungs, as detected by fluorescence imaging of mice using an In Vivo Imaging System (IVIS) Spectrum Small Animal Imaging System (PerkinElmer, Waltham, MA) as previously described^14,22^. Mice were anesthetized during fluorescence imaging in the IVIS Spectrum Small Animal Imaging System by inhalable isoflurane anesthetic. Initially, mice were anesthetized using 3.5 to 4% of inhalable isoflurane anesthetic in a closed chamber for induction of anesthesia, which took about 2 min. We then continuously applied an average of 1.5% of inhalable isoflurane anesthetic by nose cone for maintenance of anesthesia while performing the fluorescence scans on IVIS Spectrum Small Animal Imaging System. As established in our previous studies in this animal model, no metastasis to the lungs occurs prior to 15 weeks following inoculation^14^. All mice were euthanized at eight weeks following tumor inoculation, which was within the premetastatic phase prior to arrival of cancer cells in the lungs as previously established^14^. The method of euthanasia was consistent with the recommendations of the American Veterinary Medical Association (AVMA) Guidelines for the Euthanasia of Animals and was performed by intravenous injection of ketamine (20–50 mg/kg) and acepromazine (0.5–2.5 mg/kg) delivered as a 0.2 ml injection using a 26G needle in the tail vein, followed by cervical dislocation. Lungs were freeze-clamped, ground, and homogenized over liquid nitrogen. Tissue powder was subsequently weighed, aliquoted into cryotubes, and stored at -80 °C until use in future experiments including qRT-PCR and Western blotting.

### qRT-PCR

RNA isolation was performed using the RNeasy Mini Kit and QIA shredder from QIAGEN (Hilden, Germany) according to the manufacturer’s protocols. DNA digestion was performed using the RNase-Free DNase Set from QIAGEN. RNA was collected in 1.5 ml Eppendorf tubes using RNase free water at 40 °C. This slightly increased temperature allowed for maximum RNA collection off the column. 500 ng of each RNA sample was reversely transcribed to cDNA using iScript™ cDNA Synthesis Kit from Bio-Rad (Hercules, CA). The resulting cDNA was diluted tenfold. Quantitative real-time PCR (qRT-PCR) was run using iQ SYBR Green Supermix from Bio-Rad using 0.4 µl of gene-specific primer (forward and reverse combined) from Integrated DNA Technologies (Coralville, IA) and 2 µl of cDNA for each sample. qRT-PCR was performed using the iCycler RT-PCR detection system from Bio-Rad. All primers used in this study are listed in Table 1.

**Table 1:** All gene expression levels were normalized to the house keeping gene of hypoxanthine phosphoribosyltransferase (hPRT).

Gene	Species	Forward	Reverse
Col1A2	Mouse	CCAGAGTGGAACAGCGATTAC	GATGCAGGTTTCACCAGTAGAG
COL4A3	Mouse	CTGGCACTCTTAAGGTCATCTC	GTCCAGGGTCTCCTTTCATTC
COL4A5	Mouse	GACCTCCTGGGTTTGATGTT	GGTGATCCTGGAGGTCCTATAA
COL5A1	Mouse	TTACCTCCAACACCTCCAATC	GGGTCAAAGTACGGGTCATAG
COL14A1	Mouse	CTGATGTGGATTCCGGTCTATG	CAGGATCCAGGCCTTCAATAA
COL15A1	Mouse	CGTCCCTCTGGAAATGATGAA	GAGGCCAAACTCTCCTTTGT
FN1	Mouse	GTGCTTCATGCCGCTAGAT	GTGTGGATTGACCTTGGTAGAG
GPC 1	Mouse	GGAATCTGGTTTCCTCCTATGG	GCCTTCCTGGATATAGCAACTC
GPC 3	Mouse	GCAAGGAACGGGATGAAGAA	GGTTCTCAGGAGCTGGTTAATG
GPC 4	Mouse	CTAGTTTGGACCGACTGGTTAC	GCTGCCATCCTCTCATCATT
MMP1A	Mouse	CCACCTTCCTATCCTGGTAATG	TGGAGGCTCCTACCTCTAAAT
MMP1B	Mouse	GTGAGAAGAGGCTGGAGATTG	GGTTCTGGCAGGAAGCTAAT
MMP2	Mouse	GCTCTGTCCTCCTCTGTAGTTA	GGTACAGTCAGCACCTTTCTT
MMP8	Mouse	GGAAGAATCAGCAGAGGTATGG	CCTCTGCCTGGGAACTTATTG
MMP9	Mouse	CTGGAACTCACACGACATCTT	TCCACCTTGTTCACCTCATTT
MMP7	Mouse	GAGGAACACGCTGTGATAGAT	TCAGGAAGGGCGTTTGTT
MMP10	Mouse	CCCACTCTTCCTTCAGACTTAG	GCCTGCTTGGACTTCATTTC
MMP13	Mouse	GTTGACAGGCTCCGAGAAAT	CATCAGGCACTCCACATCTT
MMP14	Mouse	GCCGACTAAGCAGAAGAAAGA	GAACGGAATGTGGATTCCTAGAG
HAS2	Mouse	GTAACAGGGCCTCAGTTGTT	AGGCTTCTGTGCAGCTATTC
LOX	Mouse	CCTGGCCAGTTCAGCATATAG	GTAAGAAGTCCGATGTCCCTTG
hPRT	Mouse	CAGGGATTTGAATCACGTTTGT	TCAACAGGACTCCTCGTATTTG
Col1A1	Human	CAGACTGGCAACCTCAAGAA	CAGTGACGCTGTAGGTGAAG
Col1A2	Human	CCCAGCCAAGAACTGGTATAG	CCTTGGAAGTCACTCCTTCTAC
Col4A2	Human	CCTGGTGATGTCTGCTACTATG	GCTGATGTAGGGCTTGATCTC
Col4A3	Human	ACGGGTTCCAAAGGTGTAAG	GTACACCGACAAGTCCGTAAG
Col4A5	Human	CAGGCAGAGATGGTGATGTAG	GGGATACCTGGTTCTCCTTTG
Col5A1	Human	CGCTTACAGAGTCACCAAAGA	TCACAGTTGTTAGGATGGAGAAG
Col6A1	Human	CTCCCACCTGAAGGAGAATAAG	GCCACCGAGAAGACTTTGA
COL6A2	Human	CGTGGAGACTCAGGACAGCCA	CCTTTCAAGCCAAAGTCGCCTC
Col8A1	Human	TCCTGTACTCGGCACAAATC	GAATGCCCAAAGCAGTTAGTATG
Col11A2	Human	TGAACAGAAGGAGCTGGAATG	TGGCCTGTGAAGTCTTGATG
COL14A1	Human	CACAAACCTCCTCAGCGGAATG	GGCTTGGAGATTGGTAACACCC
Col15A1	Human	CCACCTACCGAGCATTCTTATC	CTTGAGGTTCACTATGGGAAGG
VCAN	Human	CAGCTCTTTGCTGCCTATGA	CTCCTGCCTTTCCCATCTTATC
GPC1	Human	AGAGCAGGAAGGACAGAAGA	TACTGTAAGGGCCAGGAAGA
GPC3	Human	CAGCCGAAGAAGGGAACTAAT	GTTCTTGTCCATTCCAGCAAAG
GPC4	Human	GGGAAGAGTGCCAATGAGAA	CTCTCTCTGCATAACCAGGAAC
FN	Human	AGCAGACCCAGCTTAGAGTT	GCAGAAGTGTTTGGGTGACT
MMP3	Human	CAGGCTTTCCCAAGCAAATAG	CTCCAACTGTGAAGATCCAGTAA
MMP7	Human	GTTAAACTCCCGCGTCATAGAA	GATCCTGTAGGTGACCACTTTG
MMP8	Human	AGGGAAACCAGCAACTACTC	CTTGGTCCAGTAGGTTGGATAG
MMP9	Human	GGGCTTAGATCATTCCTCAGTG	GCCATTCACGTCGTCCTTAT
MMP10	Human	CAGCGGACAAATACTGGAGAT	CTTAGGCTCAACTCCTGGAAAG
MMP13	Human	GTTTGGTCCGATGTAACTCCTC	GAAGTCGCCATGCTCCTTAAT
MMP25	Human	CAGATCAGCATGAGGACAGAAG	AACTGACAGAGGCCCAAATC
P4HA1	Human	TTGGGCAAAGTGGCCTATAC	TGGTAGAAATCTCGCCTTCATC
P4HA2	Human	ATCGTCGGATGCAGCATATC	GTCGAAGTGCGGTTCATACT
HIF1a	Human	CCAACCTCAGTGTGGGTATAAG	TTTGATGGGTGAGGAATGGG
LOX	Human	TACCCAGCCGACCAAGATA	TGGCATCAAGCAGGTCATAG
LOXL2	Human	CACTTCAGCGGGCTCTTAAA	GGAAGTCCCATGGAAGATGTG
LOXL4	Human	GAACAGCGTCTCAGGAACAA	GAAGGGCATGGCTCCAATAA

### Western blotting

Five million freshly collected cells or 30 mg of tissue were suspended in 350 $\mathrm{\mu l}$ of RIPA Buffer from Millipore Sigma (Burlington, MA) and 100X EDTA-free Halt™ Protease and Phosphatase Inhibitor Cocktail from ThermoFisher Scientific (Waltham, MA). These samples were sonicated on ice until they were completely dissolved and centrifuged at 13,000 rpm for 10 min at 4 °C. Supernatants were collected and 2 µl of supernatant was used for each data point in protein quantification using the Pierce BCA Protein Assay Kit from ThermoFisher Scientific. The remaining sample was mixed with Laemmli Sample Buffer from Bio-Rad and heated at 90 °C for 5 min. Depending on the protein probed, 20 to 30 mg of total protein from each sample underwent gel electrophoresis using 4–15% MP TGX Gel from Bio-Rad, followed by transfer onto Immun-Blot^R^ polyvinylidene difluoride (PVDF) membrane from Bio-Rad. The membrane was then blocked with 5% milk for one hour, followed by incubation with primary antibody in tris-buffered saline and polysorbate 20/tween 20 (TBST, Sigma-Aldrich, St. Louis, MO) buffer overnight at 4 °C. We used the following primary antibodies purchased from Abcam (Cambridge, UK) at the indicted dilutions in this study: Anti-collagen-1 antibody (ab21286, 1:1000), anti-collagen-4 antibody (ab19808, 1:3000), anti-MMP1 antibody (ab25483, 1:2000), anti-MMP2 antibody (ab51125, 1:1000), anti-MMP7 antibody (ab5706, 1:1000), anti-MMP8 antibody (ab53017, 1:750), anti-MMP9 antibody (ab38898, 1:3000), anti-MMP13 antibody (ab75606, 1:1000) and anti-MMP14 antibody (ab38971, 1:2000). We also purchased anti-beta-actin antibody (A5441) from Millipore Sigma (Burlington, MA) used at 1:10,000. Appropriate secondary antibodies including anti-mouse antibody (NA934VS, GE Healthcare Life Sciences, Marlborough, MA) and anti-rabbit antibody (Cell Signaling Technology, Danvers, MA) were applied to the membrane for one hour after three rounds of five-minute washing in TBST. The chemiluminescence signal from immunoreactive bands was then visualized using Pierce™ ECL Plus Western Blotting Substrate from ThermoFisher Scientific and ChemiDoc MP Imaging System from Bio-Rad. Densitometry analyses of Western Blots were performed using the Gel Analysis Tool in ImageJ (https://imagej.nih.gov/ij/)^32^.

### Statistical analysis

Differences between groups were evaluated using a two-tailed student’s t-test. p values < 0.05 were considered significant.

### Supplementary Information

Supplementary Information.

## Data Availability

All data generated and analyzed during this study are included in this published article and its Supplementary Information files.
