# Supplementary material for: Primary breast tumor induced extracellular matrix remodeling in premetastatic lungs
Source: Sci Rep. 2023 Oct 30;13:18566. doi: 10.1038/s41598-023-45832-7 (PMC10616170; doi:10.1038/s41598-023-45832-7)

**Supplementary Information for Primary Breast Tumor Induced Extracellular Matrix Remodeling in Premetastatic Lungs**

Ruoqing Cai^1^, Caitlin M. Tressler^1^, Menglin Cheng^1^, Kanchan Sonkar^1^, Zheqiong Tan^1, 5^, Santosh Kumar Paidi^2,3^, Vinay Ayyappan^1^, Ishan Barman^1,2,3^, Kristine Glunde^1,2,4,^ *

^1^The Russell H. Morgan Department of Radiology and Radiological Science, Division of Cancer Imaging Research, The Johns Hopkins University School of Medicine, Baltimore, Maryland, USA.

^2^The Sidney Kimmel Comprehensive Cancer Center, The Johns Hopkins University School of Medicine, Baltimore, Maryland, USA.

^3^Department of Mechanical Engineering, Johns Hopkins University, Baltimore, Maryland, USA.

^4^Department of Biological Chemistry, Johns Hopkins University School of Medicine, Baltimore, Maryland, USA.

^5^Department of Medical Laboratory, The Central Hospital of Wuhan, Tongji Medical College, Huazhong University of Science and Technology, Wuhan, Hubei, China

*Corresponding author:

Dr. Kristine Glunde

Department of Radiology and Radiological Science

720 Rutland Avenue

Traylor Building, Room 203

Baltimore, MD 21205

Email: kglunde1@jhmi.edu

Phone: (410) 614-2705

Fax: (410) 614-1948

Running title: ECM Changes in Premetastatic Lungs

Conflict of interest statement: The authors declare no potential conflicts of interest.

Key Words: premetastatic niche, lung, extracellular matrix, collagen, protease, degradome, breast cancer

**Supplementary Figure Legends**

**Supplementary Figure 1: Cell Culture Study.** Lung fibroblasts exposed to conditioned media from nonmalignant MCF12A breast epithelial cells, nonmetastatic MCF7 and metastatic MDA-MB-231 breast cancer cells for 24 hours. (**A**) mRNA expression levels of ECM structural proteins in lung fibroblasts. (**B**) mRNA expression levels of matrix degrading enzymes (degradome) in lung fibroblasts.

**Supplementary Figure 2: Mouse Study.** Whole body fluorescence imaging of all ten mice on IVIS Spectrum detecting tdTomato fluorescence. Tumor-bearing mice are shown in the top two rows (mouse number 6 contains a tumor, as well as a scab) and control mice are shown in the bottom row.

**Supplementary Figure 3: Mouse Study.** (**A**) mRNA expression levels of ECM structural proteins in lungs. (**B**) mRNA expression levels of LOX in lungs.

**Supplementary Figure 4: Mouse Study.** (**A**) and (**B**) show mRNA expression levels of ECM degrading enzymes in lungs.

**Supplementary Figure 5: Mouse Study.** (**A**) Protein expression levels of MMP2 in lungs. (**B**) Protein expression levels of MMP7 in lungs. (**C**) Protein expression levels of MMP14 in lungs.

**Supplementary Figure 6: Mouse Study.** Protein expression levels of collagen-1 in lungs.

**Supplementary Figure 7:** Full uncropped Western Blots from Figure 3.

**Supplementary Figure 8:** Full uncropped Western Blots from Supplementary Figure 5.

**Supplementary Figure 9:** Full uncropped Western Blots from Supplementary Figure 6.

**Supplementary Figures**

**Supplementary Figure 1**

**Supplementary Figure 2**

**Supplementary Figure 3**

**Supplementary Figure 4**

**Supplementary Figure 5**

**
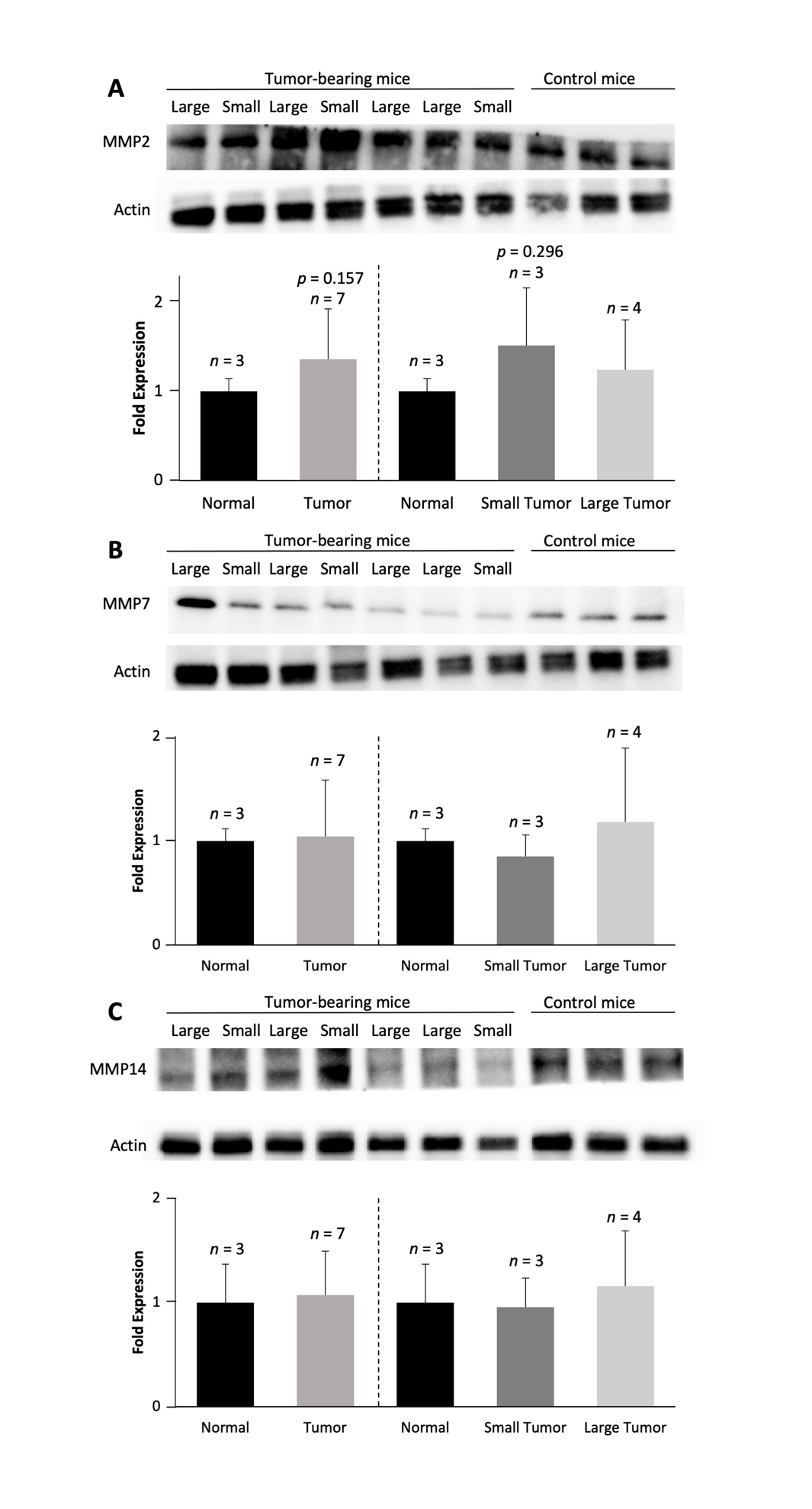
**

**Supplementary Figure 6**


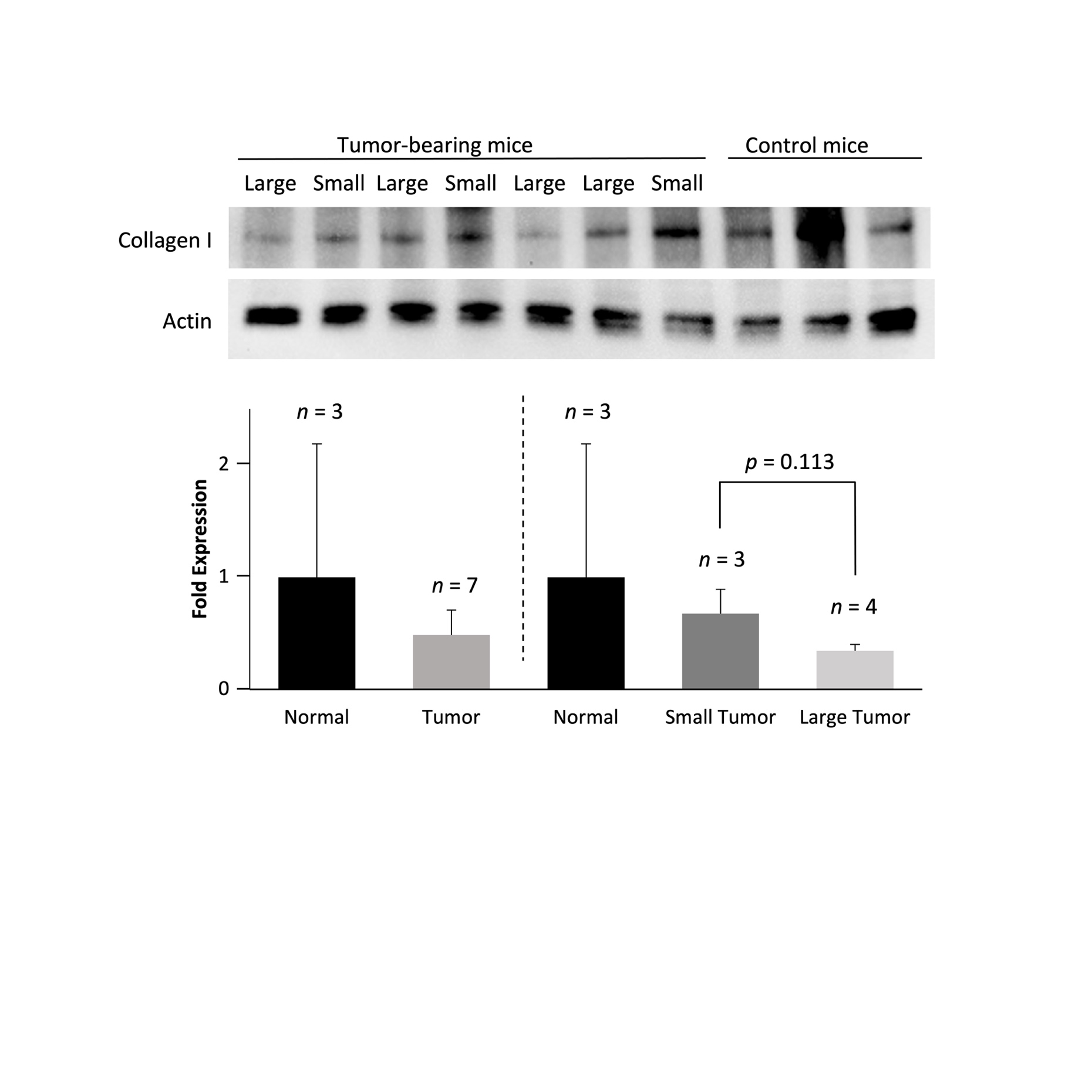


**Supplementary Figure 7**


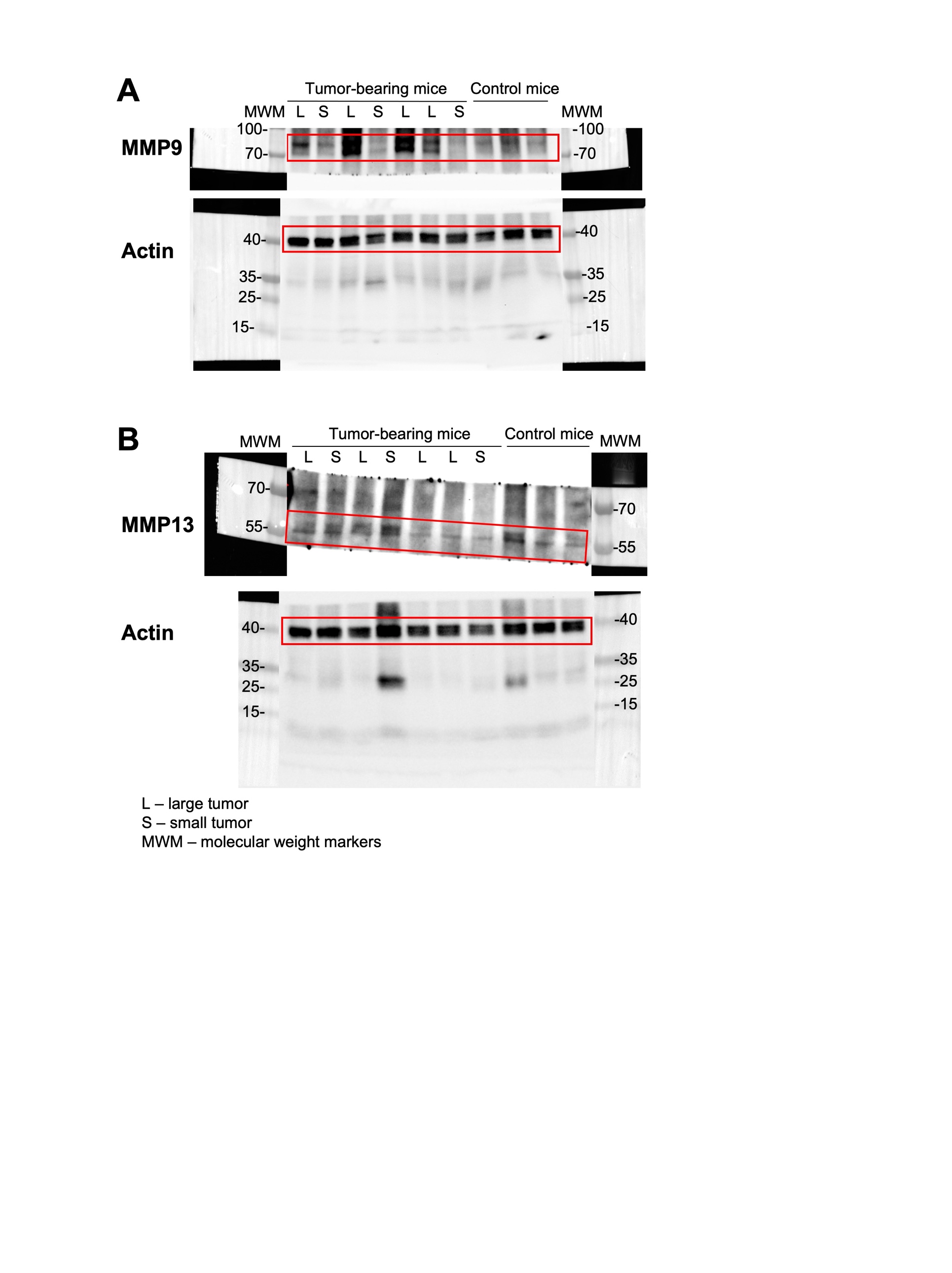


**Supplementary Figure 8**


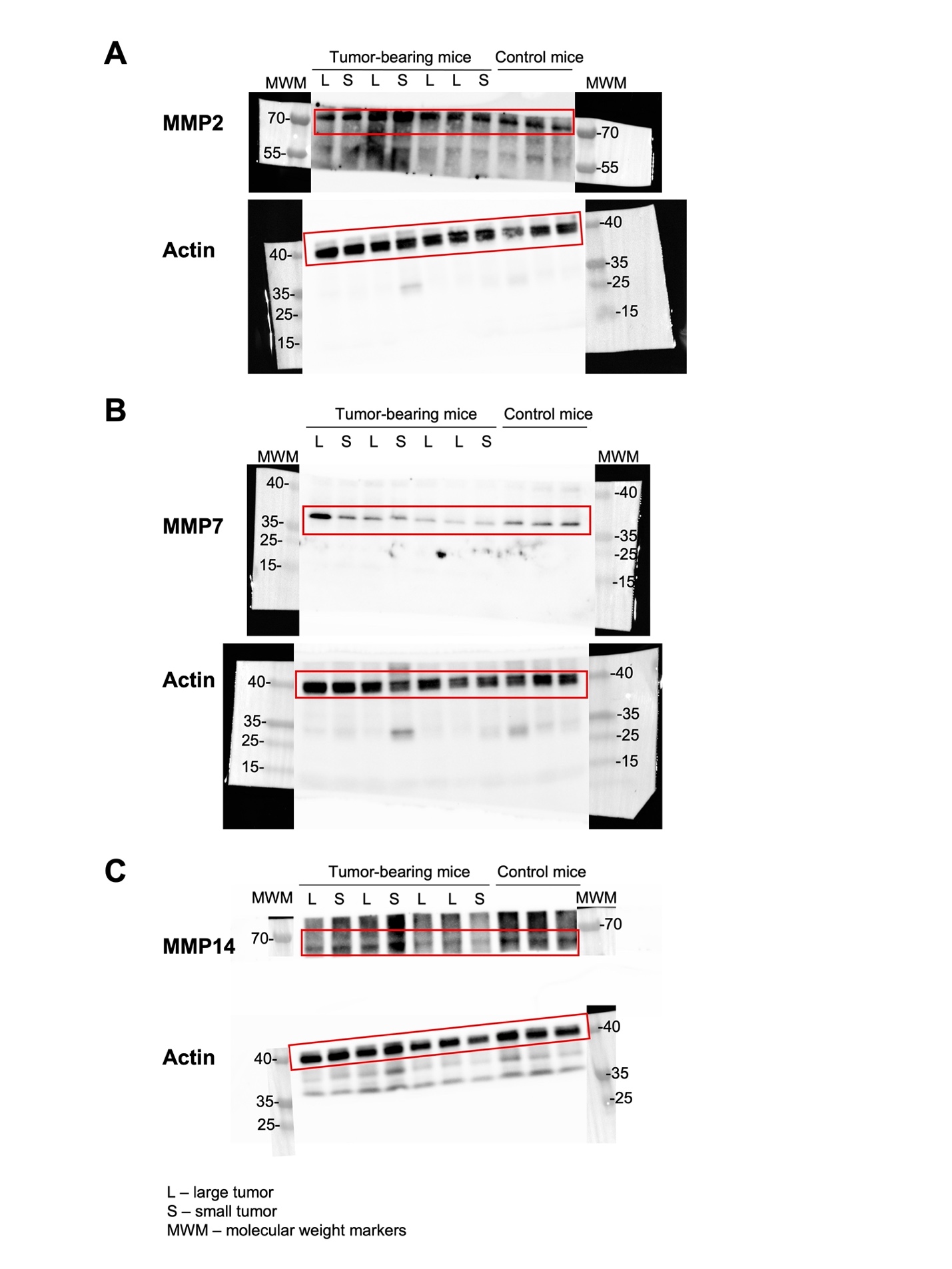


**Supplementary Figure 9**


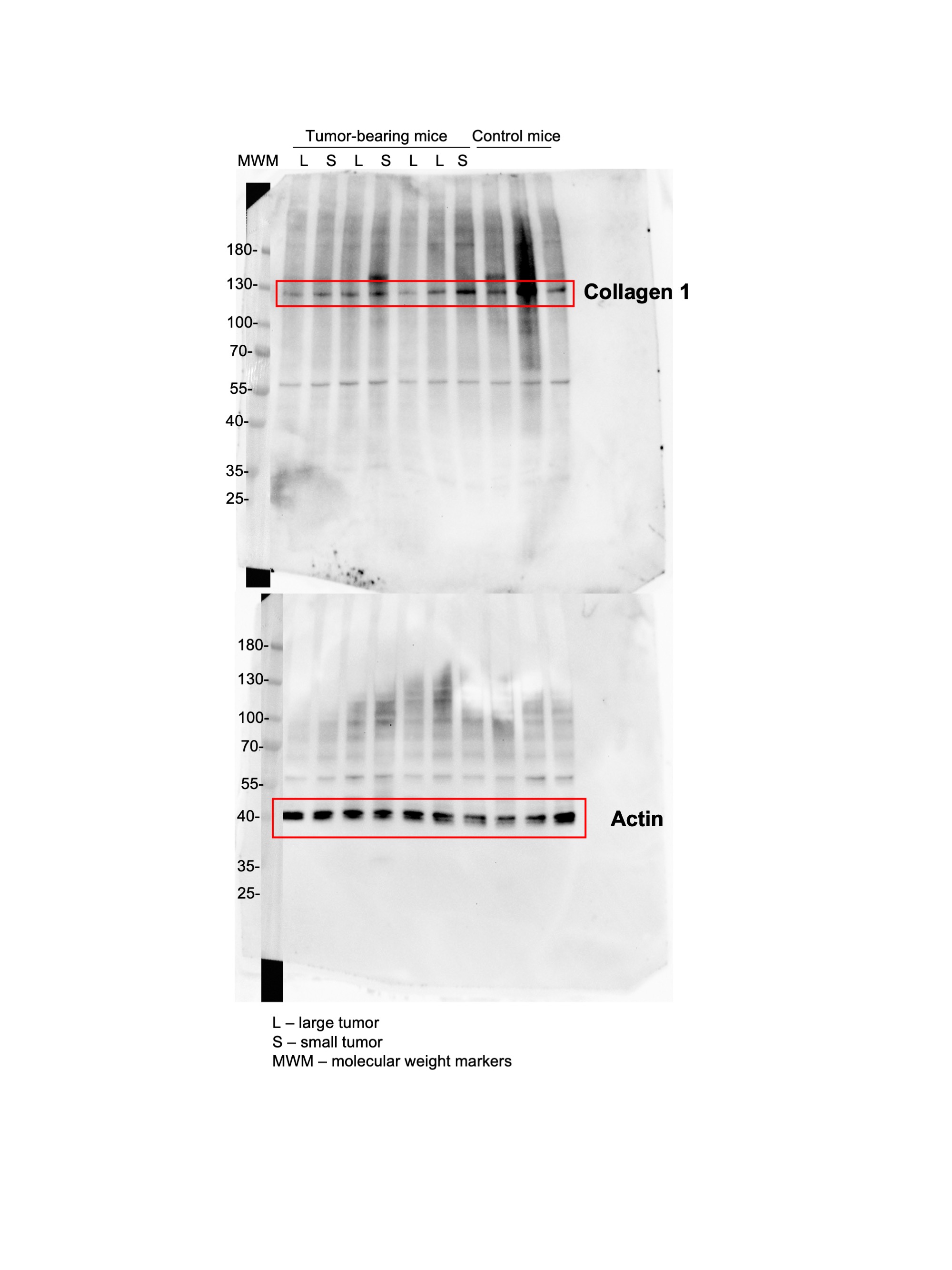

Supplement: Supplementary file 1 — Supplementary Information. [file 41598_2023_45832_MOESM1_ESM.docx]
